# A Novel Phospholipase A2 Isolated from *Palythoa caribaeorum* Possesses Neurotoxic Activity

**DOI:** 10.3390/toxins11020089

**Published:** 2019-02-01

**Authors:** Miguel Cuevas-Cruz, Fernando Lazcano-Pérez, Ulises Hernández-Guzmán, Karen Helena Díaz de la Vega-Castañeda, Sergio A. Román-González, Norma A. Valdez-Cruz, Benjamín Velasco-Bejarano, Ana Laura Colín-González, Abel Santamaría, Saúl Gómez-Manzo, Jaime Marcial-Quino, Roberto Arreguín-Espinosa

**Affiliations:** 1 Departamento de Química de Biomacromoléculas, Instituto de Química, Universidad Nacional Autónoma de México, Ciudad de México C.P. 04510, Mexico; ferlaz@hotmail.com (F.L.-P.); ulysseshrdz@gmail.com (U.H.-G.); kareninadvc@gmail.com (K.H.D.d.l.V.-C.); 2 Unidad de Proteómica, Instituto Nacional de Medicina Genómica (INMEGEN), Periférico Sur 4809, Col. Arenal Tepepan, Tlalpan, Ciudad de México 14610, Mexico; saroman@inmegen.gob.mx; 3 Programa de Investigación de Producción de Biomoléculas, Departamento de Biología Molecular y Biotecnología, Instituto de Investigaciones Biomédicas, Universidad Nacional Autónoma de México, A.P. 70228, Ciudad de México C.P. 04510, Mexico; adrivaldez1@gmail.com; 4 Sección de Química Orgánica, Departamento de Ciencias Químicas Facultad de Estudios Superiores Cuautitlán, Universidad Nacional Autónoma de México, Estado de México C.P. 54740, Mexico; qfbbevebe@gmail.com; 5 Banco de Tumores, Instituto Nacional de Cancerología, Ciudad de México C.P. 14070, Mexico; laura_coling@yahoo.com.mx; 6 Laboratorio de Aminoácidos Excitadores, Instituto Nacional de Neurología y Neurocirugía, Ciudad de México C.P. 14269, Mexico; absada@yahoo.com; 7 Laboratorio de Bioquímica Genética, Instituto Nacional de Pediatría, Secretaría de Salud, Ciudad de México C.P. 04530, Mexico; saulmanzo@ciencias.unam.mx; 8 Consejo Nacional de Ciencia y Tecnología CONACYT, Instituto Nacional de Pediatría, Secretaría de Salud, Ciudad de México C.P. 04530, Mexico; jmarcialqu@conacyt.mx

**Keywords:** cnidaria, venom, phospholipase A_2_, neurotoxin, Palythoa caribaeorum

## Abstract

Zoanthids of the genus *Palythoa* are distributed worldwide in shallow waters around coral reefs. Like all cnidarians, they possess nematocysts that contain a large diversity of toxins that paralyze their prey. This work was aimed at isolating and functionally characterizing a cnidarian neurotoxic phospholipase named A2-PLTX-Pcb1a for the first time. This phospholipase was isolated from the venomous extract of the zoanthid *Palythoa caribaeorum*. This enzyme, which is Ca^2+^-dependent, is a 149 amino acid residue protein. The analysis of the A2-PLTX-Pcb1a sequence showed neurotoxic domain similitude with other neurotoxic sPLA_2_´s, but a different catalytic histidine domain. This is remarkable, since A2-PLTX-Pcb1a displays properties like those of other known PLA_2_ enzymes.

## 1. Introduction

Zoanthids of the genus *Palythoa* inhabit the littoral zone around the globe [1]. At least 110 *Palythoa* species are cited in the literature. Like all cnidarians, zoanthids are particularly characterized by the presence of specialized cells called cnidocytes that produce stinging organelles called nematocysts. Nematocysts are a kind of ovoid capsule containing a coiled filament that when touched or chemically stimulated is projected to their possible prey with the purpose of injecting venom to paralyze them [2]. The venom includes a wide variety of toxins such as cytolysins, peptides that affect sodium [3], calcium [4], potassium channels [5], protease inhibitors [6], and phospholipases A_2_ [7], which are responsible for many harmful effects (cardiotoxicity, dermatitis, local itching, erythema, paralysis, pain, among others) [8].

Phospholipases A_2_ (PLA_2_’s) are widely distributed in different life forms such as animals [9], plants [10], bacteria [11], fungi [12], and viruses [13]. Two main families of PLA_2_’s have been characterized: (1) High 80–120 kDa cytosolic phospholipases (cPLA_2_’s) that are involved in the intracellular metabolism of arachidonic acid; and (2) 13–19 kDa secreted (sPLA_2_’s). The secreted sPLA_2_’s are characterized by two amino acid catalytic dyads (His/Asp), which vary in position depending on the type of secretory phospholipase, and require micromolar levels of Ca^2+^ for substrate-binding and catalysis [14]. The main activity of sPLA_2_ is to catalyze the S_N_2 hydrolysis of the ester bond of glycerophospholipids, and they have at least five disulfide bonds [14]. They are active in the extracellular medium and are implicated in the pathogenesis of various inflammatory processes and tumors, as well as in animal venom toxicity, mainly in bees and snakes [15]. PLA_2_’s are divided into 15 different groups. G-IA, G-IIA, G-IIB, G-III, G-IX, and G-XII PLA_2_ scaffolds have been assimilated into venoms [16]. More than 400 proteins with PLA_2_ activity have been described from animal venoms and show substantial sequence homology with each other and with mammalian PLA_2_ enzymes. Particularly, G-III PLA_2_’s have been recruited independently into four venomous lineages [17]. Many snake sPLA2’s have been well characterized structurally and have been shown to display a large diversity of activities, such as myotoxic [18], hemolytic [19], edematous [20], hypotensive [21], cardiotoxic [22], anticoagulant [23], presynaptic [24], and postsynaptic effects [25].

Neurotoxic sPLA_2_’s can induce central neurotoxicity when added to neuronal cell cultures [26,27] or during intracerebroventricular injection to animals [28,29,30]. Several PLA_2_’s display the same effect through different mechanisms [31]. Despite the fact that many studies exist about the neurotoxic activities of sPLA_2_ from animal venoms, information about the effects of sPLA_2_’s from cnidarians is scarce.

The distribution of PLA2’s among members of the phylum Cnidaria is widespread, but their enzymatic activities vary significantly between different species. Only eight PLA_2_’s have been described to date. These molecules have been isolated or cloned from the sea anemones *Condylactis gigantean* [32], *Urticina crassicornis* [33], *Bunodosoma caissarum* [8], *Adamsia carciniopados* [34], *Actinia tenebrosa* [35], and *Aiptasia pallida* [36], and the fire coral *Millepora platyphylla* [37]. Several authors have also determined PLA_2_ activity in extracts from other members of phylum Cnidaria. In addition, several predicted cnidarian PLA_2_ belonging to *Exaiptasia pallida* [38], *Nematostella vectensis* [39], *Acropora digitifera*, and *Hydra vulgaris* have been found in the NCBI and UniProt databases.

Thus, with the aim of understanding the function of sPLA_2_’s in venoms, we isolated and characterized the PLA_2_ from the zoanthid *Palythoa caribaeorum.* We found that the isolated enzyme presented a molecular mass of 16,617 Da, and exhibited neurotoxic activity in the primary motor cortex. 

## 2. Results and Discussion

Cnidarians are a diverse animal group capable of producing a vast array of molecules with different biological activities such as cytotoxic proteins, phospholipases (PLA_2_’s), hemolysins, and neurotoxic peptides. Although PLA_2_ enzymatic activity has been reported in different cnidarian tissue homogenates and is involved in the prey capture/digestion process [7], cnidarian sPLA_2_ toxin characterization has been scarcely investigated with some exceptions [8,33,34,35,36,37].

**Figure 1:**
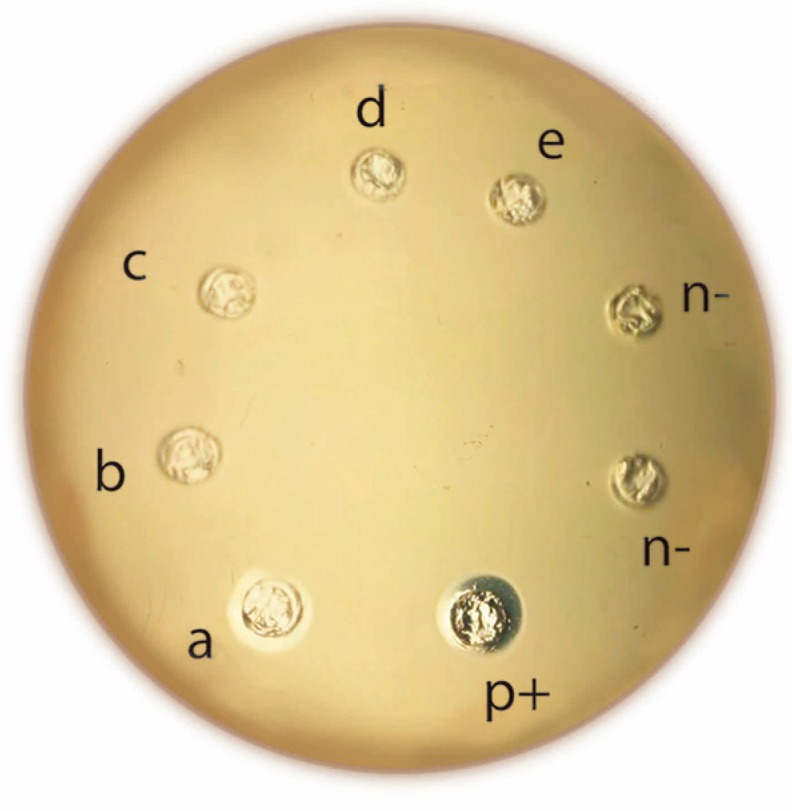
Inhibition halos of sPLA_2_ activity of total venom. The agar plate was incubated for 12 h at 37 °C. p+ (bovine pancreatic PLA_2_ as positive control); a (>30 kDa fraction) showed clear halos, which were not observed in n− (water as negative control); b (10–30 kDa fraction); c (3–10 kDa fraction), d (1–3 kDa fraction), and e (<1 kDa fraction).

**Figure 2:**
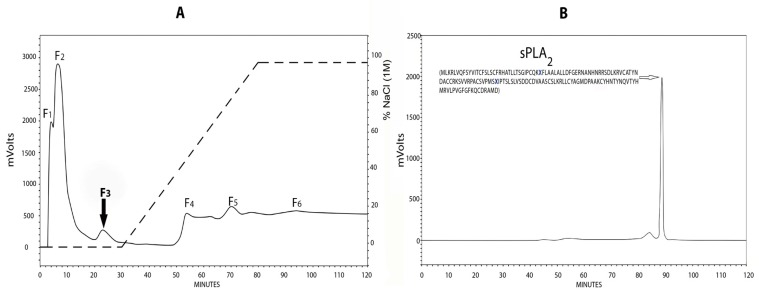
Isolation steps of PLA_2_ from *Palythoa caribaeorum*. (**A**) HPLC-cation exchange chromatography of >30 kDa fraction from *P. caribaeorum* venom. Dotted line across right-hand side of chromatogram indicates linear NaCl concentration (0–1 M) gradient used. (**B**) HPLC-size exclusion chromatography of F3. The chromatogram shows the resulting A2-PLTX-Pcb1 phospholipase A2 and its amino acid sequence (where X is no identified amino acid).

The crude venom extract fractionation by different Millipore membrane filters resulted in five fractions. The PLA_2_ activity of each fraction was determined on agar plates. Only fraction “a” showed PLA_2_ activity (Figure 1). Fraction “a” was fractionated by a cationic exchange column (Figure 2A). Six fractions were collected. Fraction 3 (F3) showed PLA_2_ activity and was subjected to a size exclusion HPLC column (Figure 2B). After these chromatographic steps, a highly pure enzyme was obtained and named A2-PLTX-Pcb1a according to the proposed nomenclature for anemone toxins [40]. The isolated PLA_2_ enzyme (A2-PLTX-Pcb1a) was analyzed by mass spectrometry. The A2-PLTX-Pcb1a amino acid sequence contains the following 149 amino acid residues: MLKRLVQFSYVITCFSLSCFRHATLLTSGIPCQKXFLAALALLDFGERNANHNRRSDLKRVCATYNDACCRKSVVRPACSVPMSXIPTSLSLVSDDCDVAASCSLKRLLCYAGMDPAAKCYHNTYNQVTYHMRVLPVGFGFKQCDRAMD (where X represents two amino acid residues that could not be determined by our method; each amino acid residue has a molecular mass of 113.18 Da, meaning that these are either a Leu or an Ile residue). Considering the literature on toxins isolated from the genus *Palythoa*, this is the first PLA_2_ isolated and characterized from this group with a determined specific activity of 13.79 meqmg^−1^min^−1^ (Table 1).

**Figure 3:**
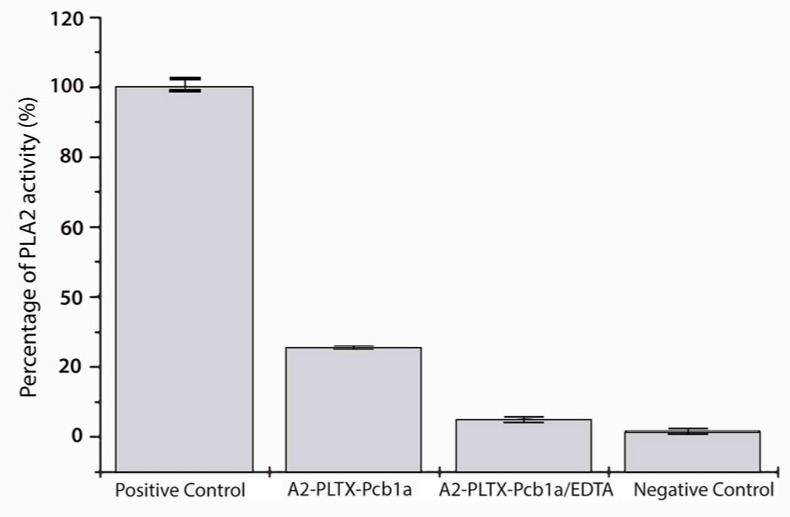
Effect of EDTA on PLA_2_ activity of A2-PLTX-Pcb1a. Data represent the mean of three independent experiments. Bee venom was used as positive control, and DTNB and assay buffer as negative control. Values represent the mean of the three replicates and standard errors are reported.

Like other phospholipase A_2_’s, A2-PLTX-Pcb1a is a calcium-dependent PLA_2_. No significant hydrolysis of DTNB by A2-PLTX-Pcb1a was detected when adding CaCl_2_ together with EDTA (Figure 3). Hence, we compared A2-PLTX-Pcb1a with other PLA_2_’s in order to find the calcium-binding site. The aforementioned site was not found in this analysis. This result suggests that, like Conodipine M, A2-PLTX-Pcb1a lacks the typical conserved calcium-binding residues present in other PLA_2_’s. Other cases of PLA_2_’s lacking this site have been reported [41]. A2-PLTX-Pcb1a displays 34.48% identity with the alpha-chain of Conodipine M from *Conus magus*, which possesses two similar domains [42]. 

The comparison of the isolated toxin sequence using clustal Ω shows that A2-PLTX-Pcb1a does not present the PLA_2_ histidine active site, due to the lack of a histidine residue in the appropriate location (data not shown). 

Likewise, the A2-PLTX-Pcb1a sequence does not present the characteristic calcium-binding region “W/Y-x-G-x-G” similarly to the members of the family IX, that includes Conodipine M [41]. The isolated PLA_2_ belongs to the sPLA_2_-type enzymes [43].

**Figure 4:**
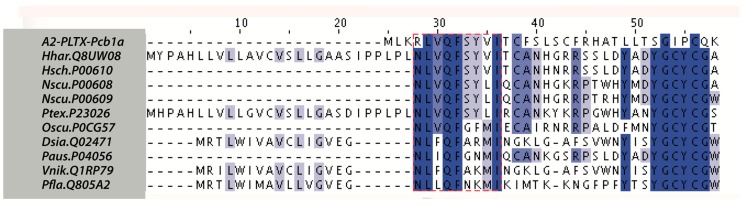
Multiple sequence alignment by Clustal Ω algorithm of neurotoxic sPLA_2_’s with A2-PLTX-Pcb1a. Neurotoxic phospholipase A_2_ (Hhar.Q8UW08, Hsch.P00610, Nscu.P00608, Nscu.P00609, Ptex.P23026, Oscu.P0CG57, Dsia.Q02471, Paus.P04056, Vnik.Q1RP79, and Pfla.Q805A2 from *Hydrophis hardwickii*, *Enhydrina schistosa*, *Notechis scutatus scutatus*, *N. s. scutatus*, *Pseudonaja textilis*, *Oxyuranus scutellatus scutellatus*, *Daboia siamensis*, *Pseudechis australis*, *Vipera nikolskii*, and *Protobothrops flavoviridis*, respectively). Conserved signature pattern, toxicity domain (NR)-L-V-Q-F-(SGAN)-X-(VLM)-I (amino acids within brackets are allowed). Identical amino acids are highlighted in blue.

**Figure 5:**
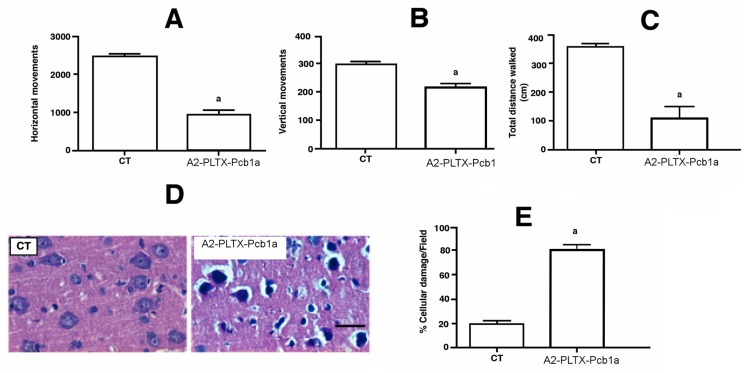
Behavioral and morphological alterations induced by an intraventricular injection of A2-PLTX-Pcb1a. Rats were administered with a single infusion of A2-PLTX-Pcb1a 3.5 mg/mL into the third ventricle. Both behavioral and morphological markers were explored seven days after A2-PLTX-Pcb1a injection. Locomotor activity parameters ((**A**) horizontal and (**B**) vertical movements as well as (**C**) total distance walked) are presented. (**D**) Histological features and (**E**) evaluation of cellular damage in primary motor cortex sections of rats infused with A2-PLTX-Pcb1a are shown. Tissue sections were histologically processed and stained with hematoxylin-eosin (×40 objectives). Mean values + S.E.M. of four experiments per group are shown, ^a^ *p* < 0.05, different from control. Student’s *t*-test.

The neurotoxin sequences analysis was performed with the Clustal Ω program using default settings and compared with homologous phospholipases. It showed a hydrophobic region of seven amino acids that is similar to a region related to the neurotoxic activity reported in various sPLA_2_ toxins (Figure 4) [44]. It has been proposed that this hydrophobic region is responsible for the PLA_2_ binding to specific membrane receptors and the initial phase of neurotoxicity [45]. To determine the neurotoxic activity of A2-PLTX-Pcb1a, the motor alterations (horizontal and vertical movements) were monitored in Wistar rats exposed to the toxin by intracerebroventricular (i.c.v.) injection. The i.c.v administration was used because the direct intracerebral administration of A2-PLTX-Pcb1a allows for the PLA_2_ toxin penetration into the cortex and periventricular areas [30]. In this animal model, it was observed that A2-PLTX-Pcb1a caused motor dysfunction in rats (Figure 5A–C). This effect has been reported for other sPLA_2_’s from other venomous animals such as snakes and bees [26]. Since the hypokinetic effects elicited by various neurotoxic sPLA_2_ have been associated with cellular damage [46,47], the cell damage (ratio of cell damage per field) throughout the different treatment groups was determined (Figure 5C). Consequently, after seven days of i.c.v. A2-PLTX-Pcb1a administration, the rat brains were collected to perform a histological analysis to determine the percentage of cellular damage (Figure 5D,E). The cerebral tissue of control rats showed a normal appearance with well-preserved cell nuclei and bodies. In contrast, treated rats showed the effects of A2-PLTX-Pcb1a on the brain, where several pyknotic nuclei could be observed (Figure 5D). Cell damage augmented in A2-PLTX-Pcb1a-treated rats as compared to the control. This effect correlates with numerous studies proposing that sPLA_2_ can elicit a β-neurotoxicity process [30,31]. Thus, A2-PLTX-Pcb1a can exert neurotoxic activity but the sPLA_2_ neurotoxicity mechanism remains to be revealed.

Cnidarian phospholipases described to date are scarce. Only two of them were isolated directly from the animal and completely sequenced. Phospholipase A2 from the sea anemone *Aiptasia pallida* consists of two isozymic forms α and β with molecular masses of 45 and 43 kDa, respectively, and are considerably larger than the typical PLA_2_’s [36]. In contrast, the PLA_2_ isolated from *Urticina crassicornis* has a molecular mass of approximately 14 kDa which corresponds with the masses reported for most PLA2’s [33]. Interestingly, both phospholipases show the enzymatic activity but produce no hemolysis when tested in erythorictes. A third known anthozoan phospholipase is that from the sea anemone *Bunodosoma caissarum* of 14.7 kDa. Hemolytic activity was not tested for this enzyme but it induced insulin secretion and kidney toxicity in rats [8]. The alignment of A2-PLTX-Pcb1a with these three toxins revealed no sequence homology (data not shown). 

**Figure 6:**
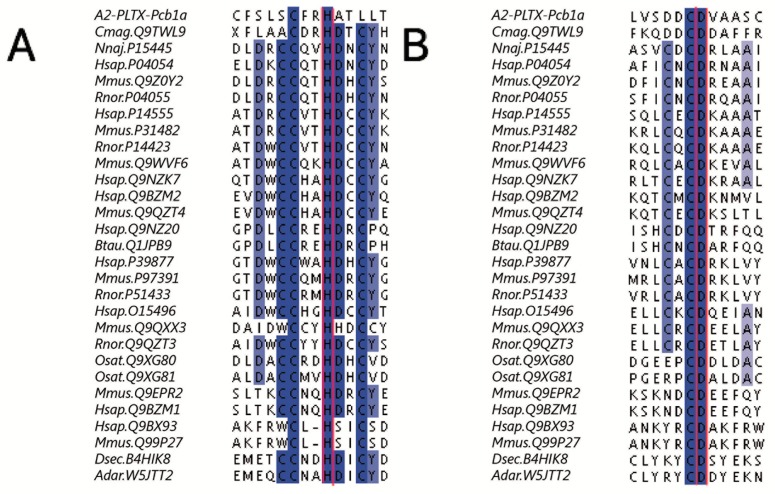
Patterns conserved of sPLA_2_ with A2-PLTX-Pcb1a were obtained from Pratt EMBL-EBI (https://www.ebi.ac.uk/Tools/pfa/pratt/). Group IX (Cmag.Q9TWL9 from *Conus magus*), Group IA (Nnaj.P15445 from *Naja naja*), group IB (Hsap.P04054, Mmus.Q9Z0Y2, Rnor.P04055 from *Homo sapiens*, *Mus musculus*, and *Rattus norvegicus*, respectively), group IIA (Hsap.P14555, Mmus.P31482, Rnor.P14423 from *H. sapiens*, *M. musculus*, and *R. norvegicus*), group IID (Mmus.Q9WVF6 from *M. musculus*), Group IIE (Q9NZK7 from *H. sapiens*), group IIF (Hsap.Q9BZM2, Mmus.Q9QZT4 from *H. sapiens* and *M. musculus*), group III (Hsap.Q9NZ20, Btau.Q1JPB9 from *H. sapiens* and *Bos taurus*), group V (Hsap.P39877, Mmus.P97391, Rnor.P51433 from *H. sapiens, M. musculus*, and *R. norvegicus*), group X (Hsap.O15496, Mmus.Q9QXX3, Rnor.Q9QZT3 from *H. sapiens, M. musculus*, and *R. norvegicus*), group XIA (Osat.Q9XG80 from *Oryza sativa japonica*), group XIB (Osat.Q9XG81 from *O. s. japonica*), group XIIA (Mmus.Q9EPR2, Hsap.Q9BZM1 from *M. musculus* and *H. sapiens*) group XIIB (Hsap.Q9BX93, Mmus.Q99P27 from *H. sapiens* and *M. musculus*), and group XIV (Dsec.B4HIK8, Adar.W5JTT2 from *Drosophila sechellia* and *Anopheles darlingi*). The common conserved signature patterns: (**A**) the “PA2_HIS (PS0018), phospholipase A2 histidine active site C-C-{P}-x-H-{LGY}-x-C” (where x represents a non-conserved amino acid, and amino acids within brackets are not allowed) and (**B**) the “PA2_ASP (PS00119), phospholipase A2 aspartic acid active site [LIVMA]-C-{LIVMFYWPCST}-C-D-{GS}-{G}-{N}-x-{S}-C” (where x represents a non-conserved amino acid, amino acids within curly brackets are not allowed and amino acids within square brackets are allowed). Identical residues are highlighted in blue.

As mentioned above, A2-PLTX-Pcb1a showed 34.48% identity with Conodipine M. And additional analysis using the Pratt EMBL-EBI tool allowed us to do a broader pattern conserved analysis of homologous sequences. After the analysis, we observed that the H22 in A2-PLTX-Pcb1a matched the H of the typical catalytic domain PS00118 PA2_His of all phospholipases (Figure 6A); additionally, the D98 of A2-PLTX-Pcb1a matched the D of the typical catalytic domain PS00119 PA2_Asp (Figure 6B).

## 3. Conclusions

Marine organisms have evolved in different physicochemical and biological environmental conditions compared to terrestrial animals, and numerous reports have shown that they could be an invaluable source of active compounds. In the present study, we purified the first PLA_2_ from *P. caribaeorum* that elicited neurotoxic activity (A2-PLTX-Pcb1a). These results open the possibility to find new sPLA_2_ structures with diverse neurotoxic mechanisms that are worthy to elucidate. In this case, due to the lack of the conserved calcium-binding residues and the low homology between A2-PLTX-Pcb1a and other reported sPLA_2_’s, it is possible that A2-PLTX-Pcb1a could represent a member of a new PLA_2_ group.

## 4. Materials and Methods 

### 4.1. Venom Extraction and Fractionation

*P. caribaeorum* specimens were collected by free diving in the La Gallega coral reef approximately 1 km off the coastline at Veracruz, Mexico. The venom was extracted by a previously described method [4]. Briefly, the material was cleaned from remnant rocks and soaked in water to eliminate the superficial mucus. To extract the nematocyst’s venom, the organisms were carefully squeezed in deionized water to expose hidden polyp tentacles and mechanically discharged. The solution was then centrifuged, lyophilized, and stored at −70 °C until use. The extract was fractionated with the ultrafiltration system (Amicon 8050 Stirred Ultrafiltration Cell 50 mL Protein Purification, Millipore, Burlington, MA, USA) using deionized water and 1, 3, 10, and 30 kDa Millipore membrane filters. The fraction that elicited phospholipase activity was subjected to a cationic exchange chromatography (HPLC TSK SP-5-PW 75 × 7.5 mm, BIO-RAD, Richmond, CA, USA) column. The ion exchange chromatography HPLC conditions included the use of buffer A (0.1 M Na_2_HPO_4_/NaH_2_PO_4_, pH 7.65) and buffer B (0.1 M Na_2_HPO_4_/NaH_2_PO_4_, pH 7.65 plus 1 M NaCl). The separation was performed at a flow rate of 0.5 mL/min. During the first 30 min, the elution was performed with buffer A, and then from 30 to 80 min using a 0–100% gradient of buffer B. At a final stage, only buffer B was used for 40 min. Proteins were monitored at 220 and 280 nm. Each fraction was collected, concentrated, and tested for phospholipase A_2_ activity. The active fractions were purified with a size exclusion HPLC TSK-gel G2000-SW column 600 × 7.5 mm (Toyo Soda, Tokyo, Japan). The size exclusion HPLC conditions included the use of deionized water at pH 7.0. The separation was performed at a flow rate of 0.15 mL/min over 120 min. 

### 4.2. Protein Determination

Protein concentration in samples was determined using the BCA^TM^ Protein Assay Kit (Thermo Scientific, Rockford, IL, USA) by comparison with bovine serum albumin (BSA) protein concentration standards [48].

### 4.3. Phospholipase Activity

#### 4.3.1. Enzymatic Activity on Agar Plates

PLA_2_ activity was measured following a modification of the protocol by Habermann and Hardt [49]. Briefly, fresh egg yolk (1 vol) and 0.85% NaCl (3 vol) were mixed and centrifuged at 2000 rpm. One milliliter of supernatant was added to 98 mL of a 0.6% agarose solution in 50 mM Tris-HCl at 50 °C (pH 7.95), followed by 1 mL of 10 mM CaCl_2_. A 15 mL portion of this mixture was poured into a Petri dish and 3-mm wells were cut in the gel. The diameters of the inhibition halos were measured after overnight incubation at room temperature.

#### 4.3.2. Enzymatic Activity on Titration Assay

PLA_2_ activity was routinely assayed at 25 °C and pH 8.9, with 2% L-α-phosphatidylcholine from egg yolk and 10 mM CaCl_2_ (Sigma-Aldrich, St. Louis, MO, USA), as described previously in the Worthington Enzyme Manual [50]. One unit of enzyme activity is defined as the uptake of NaOH in micromoles per minute. We used PLA_2_ from bovine pancreas (Sigma-Aldrich) as positive control.

### 4.4. Secretory Phospholipase A_2_ Inhibition Assay

The inhibition of PLA_2_ activity was determined using a secretory PLA_2_ colorimetric assay kit (Cayman Chemical, city, Ann Arbor, MI, USA). This assay uses the 1,2-dithio analog of diheptanoyl phosphatidylcholine as substrate and EDTA as inhibitor. Free thiols generated by PLA_2_ upon hydrolysis of the thioester bond at the sn-2 position were detected using DTNB [5,5′-dithio-bis-(2-nitrobenzoic acid)]. Color changes were monitored by a Synergy HT (BioTek, Winooski, VT, USA) microplate spectrophotometer at 414 nm, sampling 10 minutes. Ten microliters (10 μg) of bee venom PLA_2_ control was used as the reference for PLA_2_ activity, and 5 μL (500 mM) of EDTA was used in the inhibition assay. PLA_2_ inhibition was expressed in percentage of inhibition with respect to the positive control (*n* = 3).

### 4.5. Neurotoxic Effects on Rats

#### 4.5.1. Animals

All procedures with animals were strictly carried out according to the National Institutes of Health Guide for the Care and Use of Laboratory Animals, and the local guidelines on the ethical use of animals from the Ministry of Health, Mexico (NOM-062-ZOO-1999). Eight male Wistar rats (260–280 g) were used throughout the study. Animals were obtained from the vivarium of the School of Medicine from the Universidad Nacional Autónoma de México (National Autonomous University of Mexico). Rats were kept in polycarbonate cages in the same room where the immobilization protocol was performed. Animals were kept in groups of four per cage with free access to food (Laboratory rodent diet 5001; PMI Feeds Inc., Richmond, IN, USA) and water, and under controlled environmental conditions (constant room temperature (25 ± 3 °C), humidity (50 ± 10%), and light/darkness cycles (12:12 h), before the immobilization experiments began.

#### 4.5.2. Surgical Lesion Technique 

Four rats were anesthetized with sodium pentobarbital (50 mg/kg, i.p.). A single intraventricular injection of 10 µL (350 µg/mL) of A2-PLTX-Pcb1a was made with a Hamilton syringe into the third ventricle at the stereotaxic coordinates 1.3 mm posterior to bregma, 0 mm lateral to bregma and 4.6 mm ventral to the dura, according to the brain atlas in [51]. Four control animals were similarly injected with isotonic saline solution. A2-PLTX-Pcb1a and control animals were euthanized after seven days, and their brains were collected for further histological analysis.

#### 4.5.3. Motor Activity

Motor activity was estimated in all animals before they were euthanized for histological purposes in a VersaMax Animal Activity Monitor and Analyzer open field device (AccuScan Instruments, Inc., Columbus, OH, USA) for 15 min (one day before being euthanized). 

#### 4.5.4. Histology

Seven days after the intraventricular lesions, the animals were anesthetized i.p., with sodium pentobarbital and perfused transcardially with 0.9% saline solution containing heparin, followed by 4% p-formaldehyde at 4 °C. Then, brains were removed, post-fixed in 4% p-formaldehyde for 2 days, and embedded in paraffin. Fixed tissues were serially sectioned in an 820 HistoSTAT microtome (American Instrument Exchange Inc., Haverhill, MA, USA). Sections (4 μm) were stained with hematoxylin and eosin. The morphometric parameters were calculated following the “random systematic sampler”. The general criteria to score damaged neurons included pyknotic nuclei and cellular atrophy. The number of cells damaged was obtained as an average of five randomly selected fields of four sections per rat. Data were expressed as the percentage of neuronal damage per field in sections of the primary motor cortex. A 7-day period was chosen to demonstrate cell damage in the adult rat brain since this period is optimum to evidence major qualitative and quantitative morphological changes in the CNS (central nervous system) after a toxic insult. Considering that reactive gliosis after toxic insults takes 3 or 4 days to occur, and major cell loss can be observed a few days later, based on previous studies [52], the 7-day period looked adequate for this purpose. In addition, since the infusion of the protein was made in the ventricle, the time selected was propitious for the appropriate diffusion of the protein into the cortical tissue.

#### 4.5.5. Statistical Analysis

Results obtained from behavioral and morphological tests were expressed as mean values ± S.E.M. Data were analyzed by Student’s *t*-test, using the software Prism 4 (GraphPad, San Diego, CA, USA). Values of *p* < 0.05 were considered as statistically significant. 

### 4.6. Mass Spectrometry Analysis and Protein Sequencing

For mass spectrometry measurements, the purified A2-PLTX-Pcb1a was dissolved in 20% acetonitrile/0.1% TFA (triflouroacetic acid) in water. Protein solution (100 pmol/μL) was diluted 200-fold in a saturated solution of α-cyano-4-hydroxycinnamic acid, in 50% ethanol. Enzymatic digestion of A2-PLTX-Pcb1a was carried out using trypsin (modified sequencing grade). The enzyme was dissolved in 20 μL of 1 mM HCl to a final concentration of 1.25 μg/μL; an amount of 50 μL of A2-PLTX-Pcb1a protein solution (1000 pmol) was reacted with 48 μL of 25 mM NH4HCO3 at pH 8.4 and 2 μL trypsin solution. The mixture was agitated and incubated at room temperature for 3 h, and the solution was diluted 200-fold in a matrix solution. The final peptide solutions were deposited on the sample slide of a Kratos Kompact MALDI TOF-TOF (Manchester, UK) spectrometer using the Autodrop System (Microdrop, Norderstedt, Germany). The mass spectrometer was equipped with a 337 nm pulsed nitrogen laser for MALDI ionization and a curved-field reflectron for obtaining Post Source Decay spectra. The instrument was operated in the positive ion mode with 20 kV extraction voltages.

### 4.7. Sequence Analysis

A2-PLTX-Pcb1a homologous sequences selected for sequence alignment were obtained from Swiss-Prot (http://www.uniprot.org). Pairwise and multiple protein sequence alignments were generated on Jalview 2.10 software [53] with the Clustal Ω algorithm using the default parameters [54].

### 4.8. Ethical Statement 

Wistar rats were obtained from the vivarium of the School of Medicine from the Universidad Nacional Autónoma de México (National Autonomous University of Mexico). All procedures were strictly carried out according to the National Institutes of Health Guide for the Care and Use of Laboratory Animals, and the local guidelines on the ethical use of animals from the Ministry of Health, Mexico (Official Mexican Standard NOM-062-ZOO-2001). All were approved by the Ethics Committee of the National Institute of Neurology and Neurosurgery (Project code 70/15, approved in 3 March 2016). Eight male Wistar rats (260–280 g) were used throughout the study. Rats were kept in polycarbonate cages in the same room where the immobilization protocol was performed. Animals were kept in groups of four per cage with free access to food (Laboratory rodent diet 5001, PMI Feeds Inc., Richmond, IN, USA) and water, and under controlled environmental conditions (constant room temperature (25 ± 3 °C), humidity (50 ± 10%), and light/darkness cycles (12:12 h), before the immobilization experiments began.

## Figures and Tables

**Table 1:** Flow sheet of exhibited phospholipase A_2_ activity purification.

Purification Step	Protein ^a^ (mg)	Total Activity ^b^ (Units)	Specific Activity (U/mg)	Activity Recover (%)	Purification Factor
Venom	176	230.56	1.31	100	1
a	42.47	116.36	2.74	24.13	2.09
F3	3.03	16.9	5.58	1.72	4.25
A2-PLTX-Pacb1a	2.42	33.37	13.79	1.37	10.5

^a^ Proteins were estimated by BCA method. The experiments were conducted three times. ^b^ 1 Unit: mmol of fatty acid released per mg of protein using Egg-PC emulsion as substrate in the presence of 10 mM CaCl_2_. NaOH (2 mM) was used as titrating solution.
